# In Situ Formation of Ag Nanoparticles in Mesoporous TiO_2_ Films Decorated on Bamboo via Self-Sacrificing Reduction to Synthesize Nanocomposites with Efficient Antifungal Activity

**DOI:** 10.3390/ijms20215497

**Published:** 2019-11-05

**Authors:** Jingpeng Li, Minglei Su, Anke Wang, Zaixing Wu, Yuhe Chen, Daochun Qin, Zehui Jiang

**Affiliations:** 1 Key Laboratory of High Efficient Processing of Bamboo of Zhejiang Province, Engineering Technology Research Center for Building and Decorating Materials of Bamboo State Forestry Administration, China National Bamboo Research Center, Hangzhou 310012, China; lijp@caf.ac.cn (J.L.); wang_anke@126.com (A.W.); jansonwu@126.com (Z.W.); 2 International Center for Bamboo and Rattan, Beijing 100102, China; suminglei1122@163.com (M.S.); jiangzehui@icbr.ac.cn (Z.J.)

**Keywords:** bamboo, Ag/TiO_2_ nanocomposites, self-sacrificing reduction, antifungal activity

## Abstract

We developed a novel green approach for the in situ fabrication of Ag NPs in mesoporous TiO_2_ films via the bamboo self-sacrificing reduction of Ag(NH_3_)_2_^+^ ions, which can inhibit fungal growth on the bamboo surface. Mesoporous anatase TiO_2_ (MT) films were first synthesized on bamboo via a hydrothermal method. Then, Ag NPs with a 5.3 nm mean diameter were incorporated into the pore channels of optimal MT/bamboo (MTB) samples at room temperature without the addition of reducing agents, such that the Ag NPs were almost entirely embedded into the MT films. Our analysis indicated that the solubilized lignin from bamboo, which is rich in oxygen-containing functional groups, serves as a green reductant for reducing the Ag(NH_3_)_2_^+^ ions to Ag NPs. Antifungal experiments with *Trichoderma viride* under dark conditions highlighted that the antifungal activity of the Ag/MT/bamboo samples were greater than those of naked bamboo, MTB, and Ag/bamboo, suggesting that these hybrid nanomaterials produce a synergistic antifungal effect that is unrelated to photoactivity. The inhibition of *Penicillium citrinum* effectively followed a similar trend. This newly developed bamboo protection method may provide a sustainable, eco-friendly, and efficient method for enhancing the antifungal characteristics of traditional bamboo, having the potential to prolong the service life of bamboo materials, particularly under dark conditions.

## 1. Introduction

Bamboo is a widely used material in furniture, construction, and commodities trading owing to its renewability, easy processability, high strength-to-weight ratio, and negative carbon footprint [1]. However, bamboo, which is rich in nutrients, such as starch, saccharides, proteins, and aliphatics, and low in toxic constituents, is easily susceptible to attack by a variety of microorganisms such as fungi, bacteria, and insects. Such mildew-based attacks can impair its durability and cause the bamboo materials to lose their value during storage, transport, and even in their final usage [2]. Over 10% of the global annual bamboo output is damaged by microbiological attack, which greatly limits the usefulness of bamboo products, resulting in massive economic and bamboo resource losses [3]. Numerous methods have been employed to overcome this mildew problem by prolonging the service life of bamboo and adding value to bamboo products. Traditional bamboo protection methods against fungi include microwave and high-temperature treatments [4,5], which could sterilize and decrease the starch and sugar contents of the bamboo. Furthermore, chemical mold-resistant agents, including pentachlorophenol, alkaline copper quaternary compounds [6], chromated copper arsenate [7], chitosan–copper complex [8], and camphor leaf extract [9], have been used to prevent fungal growth in bamboo products. However, the use of various fungicidal chemicals has been banned or restricted owing to drawbacks such as their potential toxicity, leaching and environmental concerns, and unpleasant odor. Therefore, it is desirable to develop novel, effective, and non-toxic antifungal agents for bamboo materials.

Nanotechnological advances have permitted the development of nanosized metal oxides or their composites, such as TiO_2_ [10], ZnO [11], Fe^3+^-TiO_2_ [12], and ZnO/TiO_2_ [13,14], to mitigate fungal growth in bamboo, as outlined in our previous research. However, numerous limitations and shortcomings remain, such as the poor antifungal ability to single-phase materials, the need for ultraviolet/visible (UV/VIS) light, poor adhesion, and leaching resistance. For example, TiO_2_ coatings can combine strongly with the bamboo matrix, but single-phase TiO_2_ is not able to achieve significant fungal resistance unless it is either combined with co-biocides or in the absence of UV light. Although ZnO/TiO_2_ nanocomposites exhibit excellent antifungal activity under dark conditions, leaching resistance has become an urgent problem because nanosized ZnO was hardly incorporated into the TiO_2_ matrix that coated the bamboo substrate. Previous studies have proven that Ag nanoparticles (NPs), as well as Ag nanocomposites or Ag NP-based materials, exhibit potent antimicrobial efficacy against bacteria, viruses, and fungi with low toxicity for humans and animals [15,16,17]. The incorporation of Ag NPs into various matrices, such as cellulose-based materials [18], organic polymer complexes [19], polymer-inorganic hybrid matrices [20], and inorganic hybrid matrices [16], has recently been investigated to extend their utility in practical antimicrobial applications, particularly in inorganic hybrid matrices with mesoporous structures. Ag-containing materials that are formed by embedding Ag NPs within mesoporous nanomaterials may protect the Ag NPs from aggregation and allow for the slow release of Ag ions; as such, these materials are predicted to be more effective as an antibacterial agent than conventional Ag NPs [16,21]. Ag NPs are generally prepared via three major approaches: UV irradiation reduction [22], thermal decomposition [23], and chemical reduction [24], with chemical reduction being the most widely used method. However, this synthesis process requires the addition of reducing agents, such as sodium borohydride, hydrazine hydrate, aldehydes, or stabilizing agents, all of which have undesirable environmental impacts and require additional steps during synthesis.

The bamboo cell wall primarily comprises cellulose, hemicellulose, and lignin. Lignin is a complex phenolic polymer that comprises methoxylated phenylpropane substructures with many functional groups, such as hydroxyl, carbonyl, and aldehyde groups [25,26]; these groups can act as reductive functional groups for metal NP synthesis [27,28]. However, an exhaustive literature search indicates there is currently no report that discusses the preparation of Ag NPs by using bamboo as this reducing agent. In this study, a novel green approach was developed for the in situ fabrication of Ag NPs in mesoporous anatase TiO_2_ (MT) films via the bamboo self-sacrificing reduction of Ag(NH_3_)_2_^+^ ions. Various characterization techniques, including X-ray diffraction (XRD), Brunauer–Emmett–Teller (BET) analysis, X-ray photoelectron spectroscopy (XPS), scanning electron microscopy (SEM), high-resolution transmission electron microscopy (HRTEM), and Fourier transform infrared spectroscopy (FTIR) were employed to study surface properties such as the film microstructure, crystalline structure, surface area, and pore size. Furthermore, the mechanism for the in situ reduction of the Ag NPs in the pore channel of the MT films was investigated. This method was conducted without the use of chemical reducing or stabilizing agents such as sodium borohydride, hydrazine hydrate, and aldehydes, and could yield a highly dispersed arrangement of small Ag NPs in the MT films. These Ag-TiO_2_ composite films endowed the bamboo with excellent antifungal activity; consequently, the growth of *Trichoderma viride* (*T. viride*) and *Penicillium citrinum* (*P. citrinum*) were effectively inhibited owing to a synergistic antifungal effect that was unrelated to photoactivity. The adhesion and long-term stability of these Ag-TiO_2_ composite films on the bamboo surface were studied using Scotch tape and 2 months lab-exposure tests. Additionally, the antifungal mechanism was explored.

## 2. Results

### 2.1. Overview of Material Synthesis

**Figure 1:**
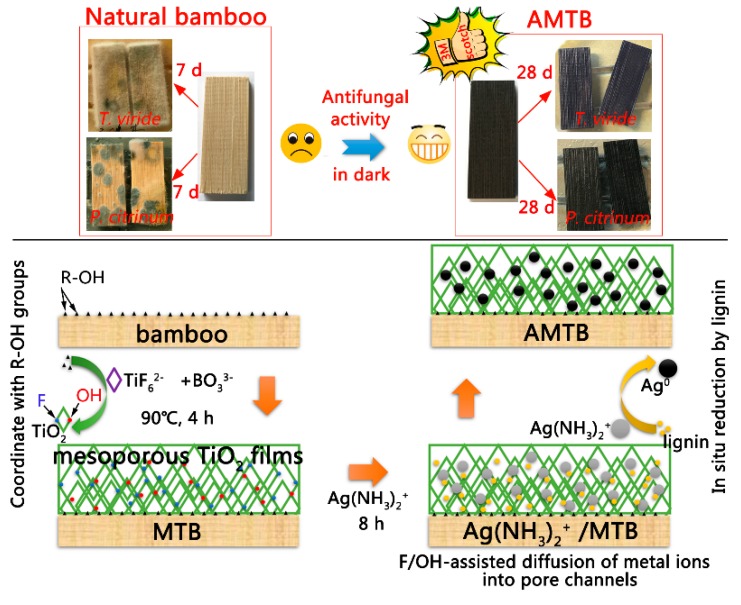
Schematic of the Ag NP-decorated mesoporous anatase TiO_2_ film-coated bamboo (AMTB) synthesis procedure and its antifungal activity owing to which fungal growth was inhibited for the two tested fungi. MTB: mesoporous anatase TiO_2_ film-coated bamboo.

The schematics of the ATMB synthesis procedure and its antifungal activity are illustrated in Figure 1. Previous studies have shown that the hydroxyl groups in the bamboo substrate can react with certain metal oxides, such as TiO_2_ [10], ZnO [11], and γ-Fe_2_O_3_ [29], and that bamboo is hydrophilic, with numerous active hydroxyl groups. The existence of numerous R–OH groups as active sites promoted the formation of R−O−Ti linkages between the bamboo surface and TiO_2_ NPs. The nucleated TiO_2_ layer on the bamboo substrate could serve as the seed layer to boost the homogeneous condensation of TiO_2_ NPs. The Ti−OH groups existing on the surface of previous TiO_2_ could further promote the growth of TiO_2_ NPs by acting as active sites for subsequent particle growth through olation and oxolation, leading to the formation of Ti−O−Ti linkages. Finally, the MT films were formed on the bamboo surface. The positively charged Ag(NH_3_)_2_^+^ was quickly drawn to the negatively charged TiO_2_ surface, which was covered by F^–^ or OH groups owing to an attractive electrostatic force. Previous studies have shown that liquid ammonia can be used for solubilizing lignin, resulting in the extraction of lignin from lignocellulose [30,31]. The metal precursors can be in situ reduced to metal NPs using various functional groups, such as the hydroxyl, carbonyl, and aldehyde groups in lignin. The MTB samples were immersed in an Ag(NH_3_)_2_OH solution, with the Ag(NH_3_)_2_^+^ ions in the Ag(NH_3_)_2_OH solution being slowly reduced by lignin and forming Ag NPs in the pore channel of MT films on the bamboo surface. The Ag NPs retained great mechanical stability even after the Scotch tape test, owing to the strong binding between the Ag NPs and TiO_2_ matrix. Fungal growth was inhibited by the strong antifungal properties of the resultant AMTB samples.

### 2.2. The Optimized Synthesis of MTB

**Figure 2:**
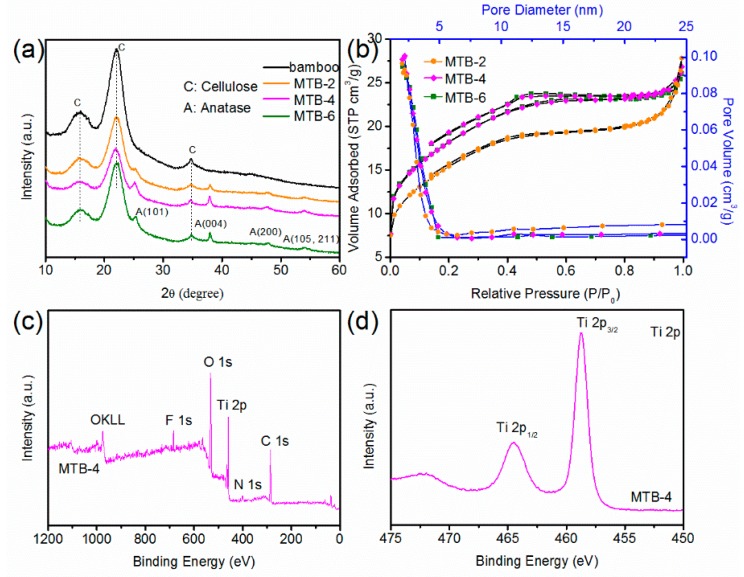
(**a**) XRD spectra of bamboo, MTB-2, MTB-4, and MTB-6. (**b**) N_2_ adsorption–desorption isotherm and pore-size distributions of MTB-2, MTB-4, and MTB-6. (**c**) X-ray photoelectron spectroscopy (XPS) survey spectra of MTB-4. (**d**) XPS spectrum of Ti 2p.

XRD and BET analyses were conducted to optimize the MTB synthesis process by determining the structural changes in the MT films on the bamboo surface at different growth times. Three characteristic broad peaks at 2θ = 16°, 22°, and 35° were associated with the crystalline diffraction of cellulose in bamboo (Figure 2a). Five new peaks were observed at 2θ = 25.3°, 37.8°, 48.0°, 53.9°, and 62.7° in the MTB samples, which were attributed to the diffraction peaks of the (101), (004), (200), (105), and (204) planes of anatase TiO_2_ structures [10]. The intensity of the anatase diffraction peaks increased as the growth time increased from 2 to 4 h; however, there was no obvious increase between 4 and 6 h. No impurity peaks were detected from this pattern, confirming that high-purity TiO_2_ films could be deposited on hydrophilic bamboo via a hydrothermal method.

The pore structure and surface area of the samples were characterized using the N_2_ adsorption–desorption isotherms (Figure 2b), with specific surface areas of 55.8, 66.4, and 65.0 m^2^ g^−1^ calculated for samples MTB-2, MTB-4, and MTB-6, respectively, by the multi-point BET method (Table 1). The pore-size analyses from the N_2_ adsorption branch revealed that the pore diameter decreased from 3.1 to 2.5 nm as the growth time increased; however, the total pore volume was not observed to change. These results indicated that the growth time of the MT films on bamboo was optimum at 4 h.

Furthermore, the chemical compositions and valence of the MTB-4 sample were confirmed via XPS analysis, with the survey spectra revealing the presence of O, Ti, F, N, and C (Figure 2c), which was consistent with previous results. It should be noted that the presence of F in MTB-4 suggests the adsorbed F^–^ ions played for a role in the formation of Ag NPs in the pore channel of the MT films. The Ti 2p_1/2_ and 2p_3/2_ core levels of MTB-4 were approximately 464.6 and 458.9 eV, respectively, as shown in Figure 2d. A binding energy difference of 5.7 eV was measured between the Ti 2p_3/2_ and 2p_1/2_ peaks of both samples, indicating that Ti was primarily in the +4-valence state (Ti^4+^).

### 2.3. Synthesis and Microstructure Characterization of AMTB

**Figure 3:**
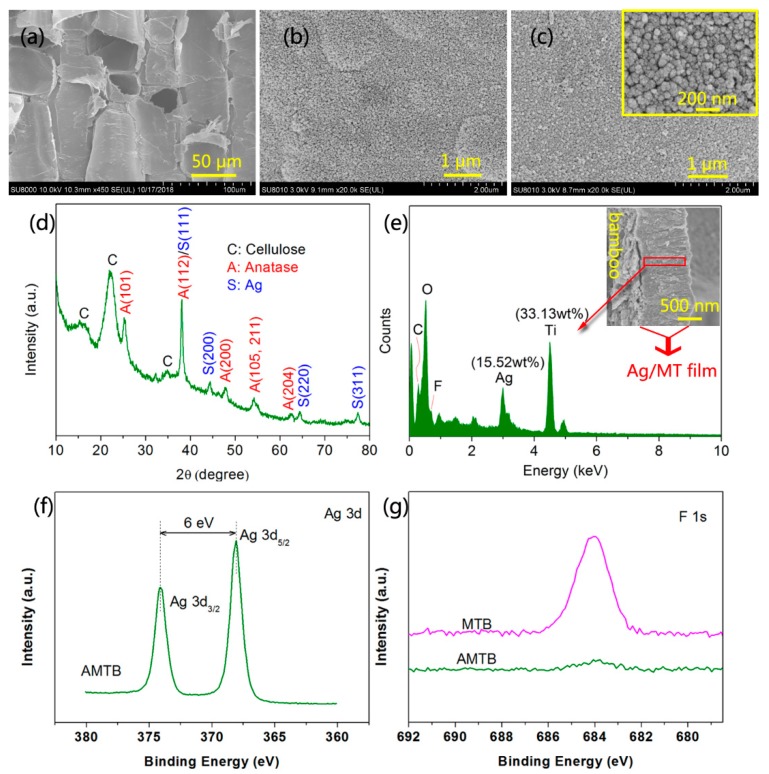
SEM images of (**a**) naked bamboo, (**b**) MTB, and (**c**) AMTB samples (The inset shows the corresponding high-magnification image of each sample). (**d**) XRD spectrum of an AMTB sample. (**e**) The energy-dispersive X-ray spectroscopy (EDS)spectrum of an AMTB sample (The inset is the cross section). (**f**) Ag 3d region of an AMTB sample. (**g**) F 1s region of the MTB and AMTB samples.

An example SEM image of naked bamboo, which comprises numerous large parenchyma cells, is shown in Figure 3a; the bamboo microstructure was the only other substance observed in the image. The MT films that were self-assembled with nanosized TiO_2_ particles were uniformly deposited on the bamboo surface after the 4 h hydrothermal reaction at 90 °C (Figure 3b). A large number of nanosized particles were observed on the anatase films, with these NPs introduced via the bamboo self-sacrificing reduction of Ag(NH_3_)_2_^+^ ions, as shown in Figure 3c. Furthermore, the structural state of the nanosized particles was characterized via XRD, with the AMTB samples exhibiting characteristic peaks that matched JCPDS card 36-1451; this indicated that the Ag NPs had face-centered cubic structures (Figure 3d) [32]. Note that the original color of the MTB samples became black–brown owing to the plasmon absorption of Ag NPs (Figure 1). Only the anatase crystal phase and cellulose were observed in the XRD spectra. Cross-sections of the AMTB samples were investigated using EDS to survey the doping depth of the Ag NPs in the MT films (Figure 3e), with the EDS results confirming the presence of Ag (15.52% Ag), indicating that numerous Ag NPs were embedded into the MT films. Furthermore, a small amount of C may have come from the bamboo component (e.g., lignin) that was solubilized in liquid ammonia and involved in the reduction of Ag(NH_3_)_2_^+^ ions. These results were supported by the FTIR analyses. XPS analysis was conducted to further determine the composition of the sample surfaces, with a narrow Ag 3d peak detected in the AMTB sample (Figure 3f), which was indicative of a major Ag^0^ component (368.2 eV for Ag^0^ 3d_5/2_ and 374.2 eV for Ag^0^ 3d_3/2_) that corresponded to the Ag NPs [33]. The F 1s peak in the AMTB sample decreased compared with that of the MTB sample owing to the consumption of F^–^ ions during the Ag NP synthesis (Figure 3g). The F^–^ ions on the TiO_2_ surface could combine with the Ag(NH_3_)_2_^+^ ions through electrostatic attraction, contributing to the formation of Ag NPs. These results indicated that MT films were successfully doped with Ag NPs.

**Figure 4:**
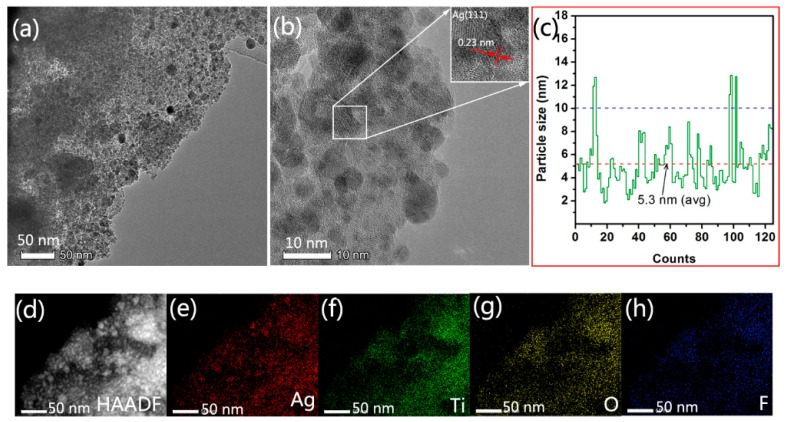
Typical (**a**) TEM and (**b**) high-resolution transmission electron microscopy (HRTEM) images, (**c**) Ag NP size distribution, (**d**) high-angle annular dark-field scanning TEM (HAADF–STEM) image, and (**e**–**h**) elemental mapping analysis of the AMTB samples.

The detailed structure of the AMTB sample and the Ag NP distribution in the MT films were further revealed via TEM, HRTEM, high-angle annular dark-field scanning TEM (HAADF–STEM), and element mapping measurements. The TEM image revealed that a high density of small particles with good dispersity was incorporated into the three-dimensional MT films (Figure 4a). The relatively dark regions in the HRTEM image were attributed to the intertwining of Ag NPs (average diameter of 5.3 nm) with the TiO_2_ films (Figure 4b). The lattice fringe spacing was 0.23 nm, which corresponded to the (111) plane of the face-centered-cubic Ag crystals. These results were consistent with the XRD results in Figure 3d. The diameter of the Ag NPs was in the 2−13 nm range (Figure 4c). The STEM analysis results are shown in Figure 4d–h. The bright spots in the HAADF image corresponded to isolated Ag atoms that remained in the MT films (Figure 4d), with a uniform distribution of Ag atoms in the AMTB sample (Figure 4e–h), which suggested that Ag NPs were successfully incorporated into the pore channels of the MT films.

### 2.4. AMTB Formation Mechanism

**Figure 5:**
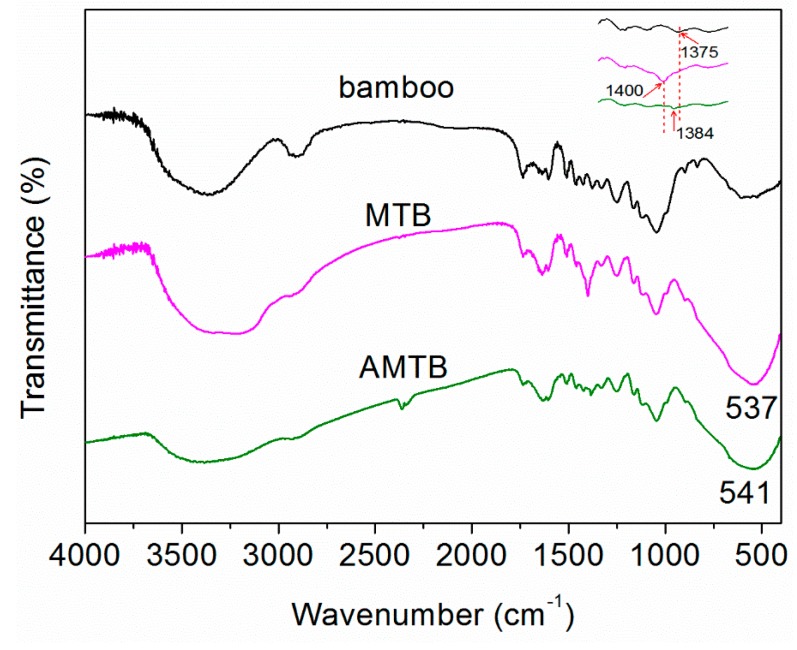
FTIR spectra of naked bamboo, MTB, and AMTB.

The FTIR spectra of naked bamboo, MTB, and AMTB were compared to investigate the AMTB formation mechanism (Figure 5). The naked bamboo absorption band at 3368 cm^−1^ was attributed to the O–H stretching vibration of the intramolecular hydrogen bond. The peak at 2912 cm^−1^ could be assigned to C–H group’s stretching vibrations. The other bands were typical bands of cellulose, hemicellulose, and lignin, as follows [11,34]: 1735 cm^−1^ for unconjugated C=O in hemicellulose, 1604 and 1508 cm^−1^ for the aromatic skeleton vibrations of lignin, 1459 cm^−1^ for the CH_3_ deformation in lignin and CH_2_ bending in xylan, 1425 cm^−1^ for the HCH and OCH in-plane bending vibrations of lignin, 1375 cm^−1^ for the CH deformation vibration of cellulose, 1330 cm^−1^ for the C−H bending of cellulose, 1250 cm^−1^ for the C−O stretching of hemicellulose and lignin, 1163 cm^−1^ for the C−O stretching of cellulose, 1046 cm^−1^ for C−O stretching, 897 cm^−1^ for C−H deformation in hemicellulose and cellulose, and 833 cm^−1^ for the benzene ring C−H bending of lignin.

A broad band was observed in the 3000–3600 cm^−1^ wavelength range of the MTB spectrum after TiO_2_ deposition, which was assigned to the stretching modes of the O−H and N−H bonds [35]. The concomitant appearance of new N−H bands at lower frequencies (3221 cm^−1^) indicated that numerous R–OH groups served as active sites and reacted with the precursor to immobilize particles on the bamboo surface. The band intensity of the C–H stretching vibration (2888–2947 cm^−1^) was significantly decreased after hydrothermal treatment. Additionally, a similar reduced band intensity was observed at 1735 cm^−1^, which corresponded to the C=O stretching vibration. Conversely, the primary bands in the MTB samples were at 537 cm^−1^, which were attributed to the Ti−O stretching and Ti−O−Ti bridging stretching modes [36]. The peak located at 1400 cm^−1^ was due to the bending vibrations of the N−H bonds in the NH_4_^+^ ions [37], which was significantly decreased after impregnation in Ag(NH_3_)_2_^+^ solution. The observed decreases in the O−H (3384 cm^−1^), C–H (2888–2947 cm^−1^), and C=O bonds (1735 cm^−1^) in the AMTB samples in combination with the existence of –CHO groups in bamboo indicated that these groups participated in the redox reaction with the Ag(NH_3_)_2_^+^ solution. These results were further supported by the FTIR spectrum of the AB sample (Figure S2). Previous studies have shown that the abundant oxygen-containing functional groups in natural cotton could be utilized to reduce silver nitrate to Ag NPs on the cotton surface [38]; furthermore, they found that the peak –OH intensity at approximately 3400 cm^−1^ decreased, indicating that some groups, such as hydroxyl, carbonyl, and aldehyde, were involved in the reduction reaction. However, the Ag(NH_3_)_2_^+^ solution in the pore channel of the MT films was not in direct contact with the bamboo substrate in our work; as such, the oxygen-containing functional groups in bamboo were not directly involved in the reduction reaction. However, the Ag(NH_3_)_2_^+^ solution was still reduced to Ag NPs in the pore channel of the MT films without adding reducing agents. A new absorption band at 1384 cm^−1^ in the AMTB spectrum was attributed to C–N group stretching vibrations. Owen et al. [31] also reported that ammonia easily penetrates into the wood structure, which causes many of the oxygen linkages that hold the cellulose and hemicellulose polymer chains together to break down. The isolated lignin could react with ammonia to form ammonium salts. A typical lignin UV spectrum was observed at 294 nm, indicating that some lignin was solubilized in ammonia solution, as shown in Figure S3. [39] These ammonium salts in solution could potentially play a crucial role in reducing the Ag(NH_3_)_2_^+^ ions to Ag NPs in the pore channel of the MT films. The AMTB sample was successfully fabricated via the direct bamboo self-sacrificing reduction of Ag(NH_3_)_2_^+^ ions in the TiO_2_ matrix.

### 2.5. Antifungal Activity of AMTB

#### 2.5.1. Inhibition of *T. viride* Spores

**Figure 6:**
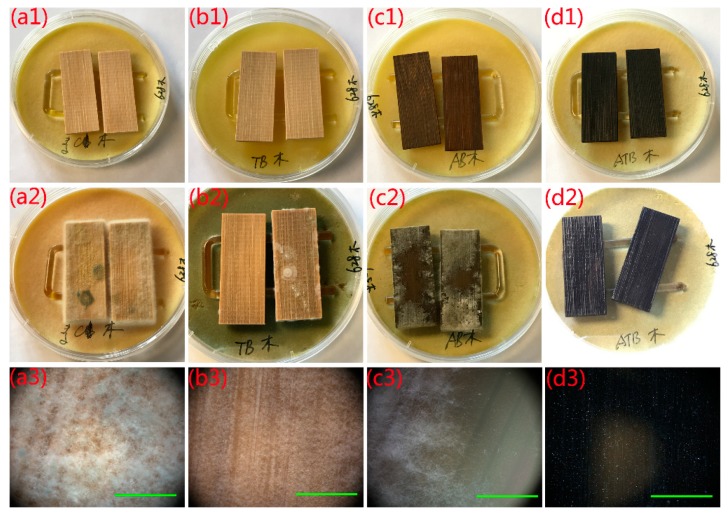
Antifungal properties of (**a1–a3**) naked bamboo, (**b1–b3**) MTB, (**c1–c3**) Ag/bamboo (AB), and (**d1–d3**) AMTB to inhibit *Trichoderma viride* growth. Incubation period: (**a1–d1**) 0 days, (**a2**) 7 days, (**b2–d2**) 28 days. The optical microscope images in (**a3–d3**) correspond to the samples in (**a2–d2**). Scale bars: 1 mm.

The antifungal activity of naked bamboo, MTB, AB, and AMTB and their ability to inhibit *T. viride* spores are shown in Figure 6. The test specimens were supported by a U-shaped glass rod (4 mm diameter) on the mycelia-covered PDA substrates and had no direct contact with the spores. We can clearly see that the naked bamboo was entirely covered with mycelia after incubation for 7 days (Figure 6a2,a3), indicating that naked bamboo had no resistance to *T. viride*. A small number of mycelia were directly observed on the MTB sample surface after incubation for 28 days (Figure 6b2), with its optical microscope image illustrating that it was completely covered with mycelia (Figure 6b3), similar to that observed for natural bamboo. However, the fungal growth was much more robust in the naked bamboo. Mycelia can grow well in the bamboo in the AB sample after incubation for 28 days, even though many nanosized Ag particles were coated on the bamboo surface (Figure 6c2,c3). These results indicated that the AB samples possessed a poor resistance to *T. viride.* Both the MTB and AB samples had very limited antifungal activity under dark conditions. Conversely, we did not observe mycelia on the AMTB sample surface (Figure 6d2,d3), which indicated that the antifungal activity of the AMTB sample was greater than that of both the MTB and AB samples. These observations suggest that the Ag-TiO_2_ hybrid materials produce a synergistic antifungal effect that is unrelated to photoactivity.

#### 2.5.2. Inhibition of *P. citrinum* Spores

**Figure 7:**
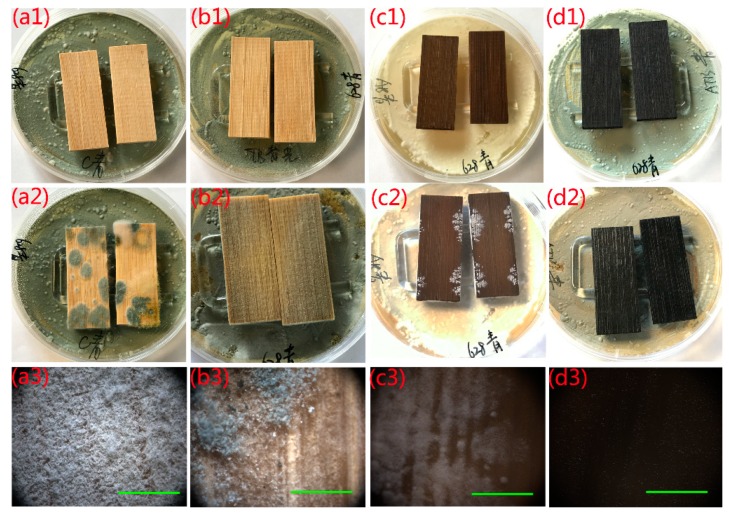
Antifungal properties of (**a1–a3**) naked bamboo, (**b1–b3**) MTB, (**c1–c3**) AB, and (**d1–d3**) AMTB to inhibit *Penicillium citrinum* growth. Incubation period: (**a1–d1**) 0 days, (**a2**) 7 days, (**b2–d2**) 28 days. The corresponding optical microscope images in (**a3–d3**) correspond to the samples in (**a2–d2**). Scale bars: 1 mm.

*P. citrinum* was also chosen to confirm the aforementioned conclusion and verify the antifungal activity of the as-prepared samples in the same assays. Multiple fungus clusters were observed on the surface of the naked bamboo after incubation for 7 days (Figure 7a2,a3). The bamboo surface was almost entirely covered with mycelia in the optical microscope image, indicating that the natural bamboo had no resistance to *P. citrinum*. The MTB sample also had poor resistance to *P. citrinum* (Figure 7b2,b3) after incubation for 28 days, similar to its poor resistance to *T. viride.* However, the AB sample showed better antifungal activity than the naked bamboo and MTB samples, as *P. citrinum* mycelia failed to cover the entire surface of the AB sample after incubation for 28 days (Figure 7c2,c3). The area of fungal infection reached 35% (average) at the end of the 28 days incubation period. This also indicated that the antifungal activity of the AB samples for *P. citrinum* was more effective than that for *T. viride*. The AMTB sample exhibited efficient antifungal activity for *P. citrinum* after incubation for 28 days in dark conditions, with no mycelia observed on the AMTB sample surface (Figure 7d2,d3).

**Figure 8:**
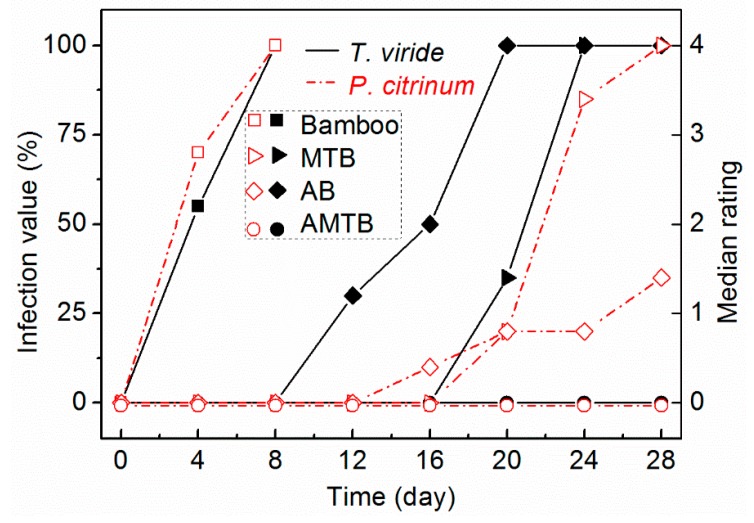
Fungal growth curves for two fungal species (*T. viride* and *P. citrinum*) cultured on naked bamboo, MTB, AB, and AMTB.

The antifungal activity of the naked bamboo, MTB, AB, and AMTB samples during the 28 days incubation period are shown in Figure 8. The naked bamboo was seriously infected with both *T. viride* and *P. citrinum*, with the surface infection value reaching ratings of 2.2 and 2.8 on day 4, and 4 and 4 on day 8, respectively. There was a marked improvement in the antifungal activity of the MTB sample against *T. viride* and *P. citrinum* compared with that for naked bamboo. The fungal growth on the MTB surface was slower than that on the naked bamboo surface, with the surface infection value of the MTB sample possessing a 0 rating after 14 days. However, fungi spores began to germinate and grew rapidly after 14 days, with surface infection values reaching ratings of 4 (*T. viride*) and 3.4 (*P. citrinum*) on day 24. The AB sample initially became infected with *T. viride* and *P. citrinum* on days 8 and 12, respectively. The surface infection value for *T. viride* reached a rating of 4 on day 20, whereas it only reached 1.4 for *P. citrinum* at the end of the experiment. The antifungal activity of the AB samples to *P. citrinum* was therefore more effective than that to *T. viride*. Conversely, the surface infection values of the AMTB samples possessed 0 ratings for both *T. viride* and *P. citrinum* at the end of the experiment, indicating that the AMTB samples displayed effective resistance to fungal growth.

### 2.6. Exploration of the Antifungal Mechanism

Recent studies have indicated that the mechanism responsible for the activation of the biocidal properties of Ag/TiO_2_ nanocomposites was rather complicated. The reasons for the enhanced antimicrobial effect of Ag/TiO_2_ hybrids in the absence of UV light are still not completely understood. Their enhanced antimicrobial qualities originated from the light-mediated generation of reactive oxygen species, release of toxic silver ions, and cell membrane damage through their contact with the Ag NPs. Li et al. [40] reported that hybrid Ag/TiO_2_ nanocomposites possessed stronger bactericidal activity than pure Ag and pure TiO_2_ under UV light. It is well known that the doping of Ag NPs on TiO_2_ enhances the photocatalytic activity of TiO_2_, resulting in the enhanced generation of reactive oxygen species. They also suggested that the release of Ag^+^ ions was not the dominant inactivation mechanism for the Ag/TiO_2_ nanocomposites [40]. Jin et al. [41] reported a similar observation, where the Ag^+^ ions released from Ag_2_O/TNBs did not contribute to the bactericidal effects of Ag_2_O/TNBs in dark conditions. However, another study also showed that the Ag^+^ ion release rates from the Ag/TiO_2_ nanocomposites were much higher than those from the Ag NPs, resulting in better bactericidal activity [42]. Furthermore, previous studies have suggested that the enhanced antimicrobial activity of nanocomposites may be due to their large surface-to-volume ratio. However, the BET surface area of the TiO_2_ particles decreased owing to Ag NP modification (Table S1). A comparison of the BET surface area and antifungal activity suggested that the surface area was not the main factor contributing to the enhanced antifungal effect. Perkas et al. [43] reported that nanocomposites with smaller-sized Ag NPs incorporated in titania possessed higher antibacterial properties. Esfandiari et al. [44] reported a similar observation, noting that the bactericidal capacity was dependent on the size characteristics of the Ag/TiO_2_ coating. Here, numerous Ag NPs that were 50–100 nm in diameter were similarly prepared on the MTB surface via a silver-mirror reaction (Figure S4a). Some *T. viride* mycelia were observed on its surface after incubation for 28 days (Figure S4c), revealing a much poorer antifungal activity than that of the AMTB samples. We believe this could be attributed to the small particle size of the self-sacrificing reduction-derived AMTB (2−10 nm) compared with that of the silver-mirror-reaction-derived AMTB (50−100 nm). The ATMB antifungal activity was greater than those for the MTB and AB samples in the absence of light, suggesting that the Ag/TiO_2_ hybrid materials produced a synergistic antifungal effect that was unrelated to photoactivity.

### 2.7. Stability Evaluation

**Figure 9:**
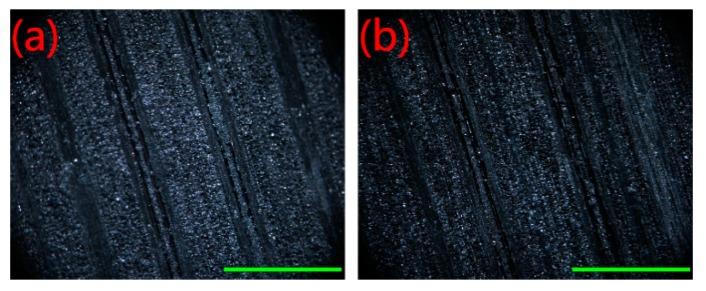
Optical microscope images of the AMTB surfaces during the Scotch tape tests: (**a**) original surface; (**b**) after 15 peeling tests. Scale bars: 1 mm.

The Scotch tape test, which was based on ASTM D3359-02 standard, was applied to determine the stability and durability of the Ag-incorporated MT films on the bamboo surface. Scotch tape was pressed against the AMTB sample and subsequently peeled off. Optical images of the AMTB surface before and after 15 peeling attempts are shown in Figure 9, with no obvious damage or detachment of the films after the test. However, slight black particles were observed on the 3M Scotch tape after the first peeling attempt (Figure S5), but this did not affect the antifungal activity of the test AMTB samples. No mycelia were observed on the AMTB surface after incubation for 28 days, indicating the films had good adhesion after the Scotch tape test. Furthermore, the antifungal activity of the AMTB samples after exposure in the lab environment for two months was measured to determine the long-term stability of the AMTB samples. Mycelia failed to cover the AMTB sample surface after incubation for 28 days, indicating that the AMTB samples also retained good long-term stability.

## 3. Conclusions

We have introduced a simple, sustainable, and environmentally friendly method for the in situ fabrication of Ag NPs into mesoporous TiO_2_ films via bamboo self-sacrificing reduction. The mesoporous anatase TiO_2_ films provided sufficient active sites (F or OH groups) for the Ag(NH_3_)_2_^+^ ions diffusing into its pore channels. The solubilized lignin from bamboo, which is rich in oxygen-containing functional groups, served as a green reductant for reducing the Ag(NH_3_)_2_^+^ to Ag NPs in the pore channels. Natural bamboo was not only used as a reductant to nucleate the Ag precursors but also as a support to immobilize the Ag-TiO_2_ composite films. These Ag-TiO_2_ composite films endowed the bamboo with excellent antifungal activity with which *T. viride* and *P. citrinum* was inhibited, with this synergistic antifungal effect being unrelated to photoactivity. Furthermore, the high antifungal activity was found to be dependent on the size of the Ag NPs. Moreover, the Scotch tape and 2 months lab-exposure tests indicated that the Ag-TiO_2_ composite films on the bamboo surface had good adhesion and long-term stability. The use of bamboo as an environmentally friendly and sustainable material with abundant functional groups could serve as a general support to produce metal/bamboo functional materials for a broader range of catalytic and environmental remediation applications.

## 4. Materials and Methods

### 4.1. Materials

Air-dried moso bamboo (*Phyllostachys edulis* (Carr.) J.Houz.) specimens (50 (longitudinal) × 20 (tangential) × 5 mm (radial)) were derived from Zhejiang YoYu Corporation (Anji, China). Ammonium hexafluorotitanate ((NH_4_)_2_TiF_6_), boracic acid (H_3_BO_3_), silver nitrate (AgNO_3_), and ammonia solution (NH_4_OH, 25%–28%) were purchased from Aladdin Chemistry Co., Ltd. (Shanghai, China). All the chemicals used in this experiment were of analytical reagent grade. Potato dextrose agar (PDA; 1 L of water, 6 g of potato, 20 g of dextrose, and 20 g of agar, pH = 5.6) was obtained from Qingdao Hope Bio-Technology Co., Ltd. (Qingdao, China). Deionized water (DI water) was prepared with a Milli-Q Advantage A10 water purification system (Millipore, Bedford, MA, USA) and used throughout the experiments.

### 4.2. Preparation of the Ag NP-Decorated MT Film-Coated Bamboo (AMTB) Samples

The MT films were synthesized on the bamboo surface through a modified procedure outlined in our previous work [13]. A (NH_4_)_2_TiF_6_ and H_3_BO_3_ solution was first mixed and transferred into a 50 mL Teflon-lined autoclave that contained a bamboo specimen without pH adjustment, and then oven-heated at 90 °C for 2, 4, and 6 h. The resultant MT film-coated bamboo (MTB) samples were then dried overnight at 60 °C. Ag NPs were embedded into the MTB samples by immersing the dried MTB samples in 0.1 M Ag(NH_3_)_2_OH solution for 8 h at room temperature using fresh Ag(NH_3_)_2_OH solution that was prepared via the dropwise addition of ammonia solution into aqueous AgNO_3_ solution until the brown precipitate was dissolved, producing a clear solution. The impregnation of Ag NPs onto the MTB sample surface was deemed successful when the sample turned black–brown. Finally, the resultant Ag NP-decorated MTB (AMTB) samples were washed repeatedly with DI water and oven-dried at 50 °C for 24 h. Ag/bamboo (AB) samples were also prepared following the same Ag impregnation procedure.

### 4.3. Characterization

XRD patterns were acquired using a Bruker AXS D8 Advance diffractometer (Bruker, Billerica, MA, USA) with a Cu Kα (1.5406 Å) radiation source that operated at 40 kV voltage and 40 mA current. The BET surface areas were measured from the N_2_ adsorption–desorption isotherms that were acquired using a Micromeritics ASAP 2020 surface analyzer (Micromeritics, Norcross, GA, USA), with the pore-size distribution curves calculated from the adsorption branch of the isotherm. XPS measurements were acquired using a Thermo ESCALAB 250Xi spectrometer (Thermo Scientific, Waltham, MA, USA) with an Al Kα X-ray source. SEM images and EDS spectra were obtained using a field-emission SEM (Hitachi SU8010, Tokyo, Japan). Structural analysis was conducted via TEM/HRTEM (TF20, Jeol 2100F, 200kV; JEOL, Tokyo, Japan). FTIR analysis was conducted using an FTIR spectrometer (Nicolet Magna 550; GMI, Ramsey, MN, USA) in the 4000–400 cm^−1^ range with KBr pellets. The UV/VIS diffuse reflectance spectra were recorded using a UV-2550 spectrophotometer (Shimadzu, Japan) in the 250–600 nm range.

### 4.4. Antifungal Test

The antifungal tests of the as-prepared samples were conducted on the basis of Chinese Standard GB/T 18261-2013. *T. viride* and *P. citrinum* were used during all the experiments because they are common fungi that are found in infected bamboo. The fungi spores were obtained from the BeNa Culture Collection (BNCC, Beijing, China) and needed to be activated before use. The activated fungi spores with approximately 1 × 10^6^ CFU/mL (CFU: colony forming unit) were inoculated onto each PDA plate at 25 °C and 95% relative humidity for 7 days until sporulation. The as-prepared samples and U-shaped glass rod was sterilized using an autoclave steam sterilizer at 121 °C and 0.1 MPa for 30 min (MLS-3750; Sanyo, Osaka, Japan) prior to inoculation. A sterilized U-shape glass rod (4 mm diameter) was placed on the PDA substrate, which was covered with mycelium, and two specimens were placed separately onto the glass rod, as shown in Figure S1. Then, the dishes were placed into a climate chamber (BIC-400; Boxun, Shanghai, China), where temperature and relative humidity were fixed at 25 °C and 95%, respectively. The tests were conducted for 28 days. The as-prepared samples (bamboo, MTB, AB, and AMTB) were used for the antifungal tests in the absence of light irradiation. All of the experiments were performed in sextuplet, with the mean values provided in the paper. The fungi control effectiveness was calculated as follows: 0 rating indicated no fungal growth on the sample surface, 1 indicated a surface infection area of less than one-quarter, 2 indicated a surface infection area between one-quarter and one-half, 3 indicated a surface infection area between one-half and three-quarters, and 4 indicated a surface infection area of greater than three-quarters. Lower infection values represented better antifungal treatment and vice versa.

### 4.5. Stability Evaluation

The mechanical durability and long-term stability of the ATMB samples were conducted via the Scotch tape test, which was based on ASTM D3359-02 standard, and the 2 months exposure test in a lab environment. Scotch tape was pressed against the AMTB substrate and subsequently peeled off, with the peeling test repeated up to 15 times. Another group of AMTB samples was exposed in the lab environment for 2 months. Then, these two groups of samples were incubated in a climate chamber for 28 days at 25 °C and 95% relative humidity in dark conditions.

## Figures and Tables

**Table 1:** Summary of the surface area and pore properties of the MTB samples.

Sample	*S*_BET_/m^2^ g^−1^	*d*_P_/nm	*V*_P_/cm^3^ g^−1^
MTB-2	55.8	3.1	0.04
MTB-4	65.4	2.5	0.04
MTB-6	65.0	2.5	0.04

## Supplementary Materials

The following are available online at https://www.mdpi.com/1422-0067/20/21/5497/s1.
