# Supplementary material for: In Situ Formation of Ag Nanoparticles in Mesoporous TiO2 Films Decorated on Bamboo via Self-Sacrificing Reduction to Synthesize Nanocomposites with Efficient Antifungal Activity
Source: Int J Mol Sci. 2019 Nov 5;20(21):5497. doi: 10.3390/ijms20215497 (PMC6862692; doi:10.3390/ijms20215497)
Supplement: Supplementary file 1 [file ijms-20-05497-s001.pdf]

International Journal of
Molecular Sciences

MDPI

Supplementary Materials

In-situ formation of Ag nanoparticles in mesoporous TiO_2 films decorated on bamboo via self-sacrificing reduction to synthesize nanocomposites with efficient antifungal activity

Jingpeng Li [1], Minglei Su [2], Anke Wang [1], Zaixing Wu [1], Yuhe Chen [1,*], Daochun Qin [2,*] and Zehui Jiang [2]

[1] Key Laboratory of High Efficient Processing of Bamboo of Zhejiang Province, Engineering Technology Research Center for Building and Decorating Materials of Bamboo State Forestry Administration, China National Bamboo Research Center, Hangzhou 310012, PR China; lijp@caf.ac.cn (J.L.); wang_anke@126.com (A.W.); jansonwu@126.com (Z.W.); yuhec@sina.com (Y.C.)

[2] International Center for Bamboo and Rattan, Beijing 100102, PR China; suminglei1122@163.com (M.S.); qindc@icbr.ac.cn (D.Q.); jiangzehui@icbr.ac.cn (Z.J.)

* Correspondence: yuhec@sina.com (Y.C.); qindc@icbr.ac.cn (D.Q.)

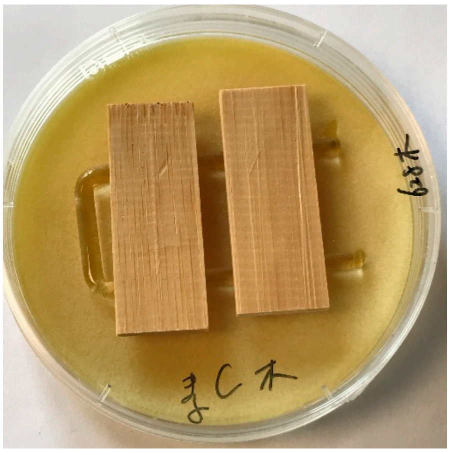

Figure S1. Method for the antifungal test.

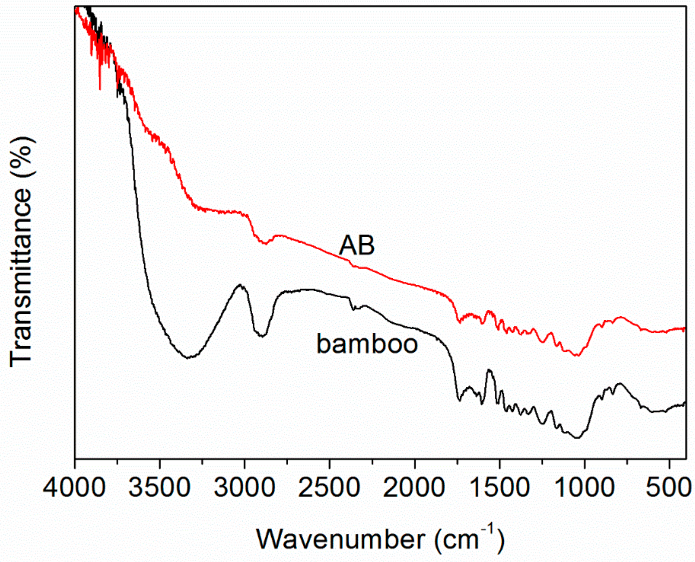

Figure S2. FTIR spectra of naked bamboo and AB samples.

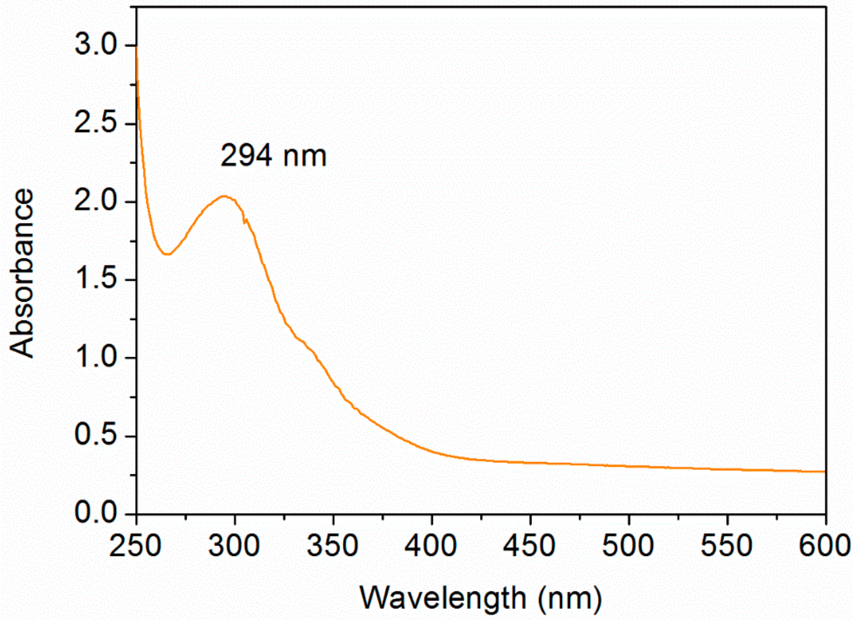

Figure S3. UV-vis spectrum of lignin in the reaction mixture.

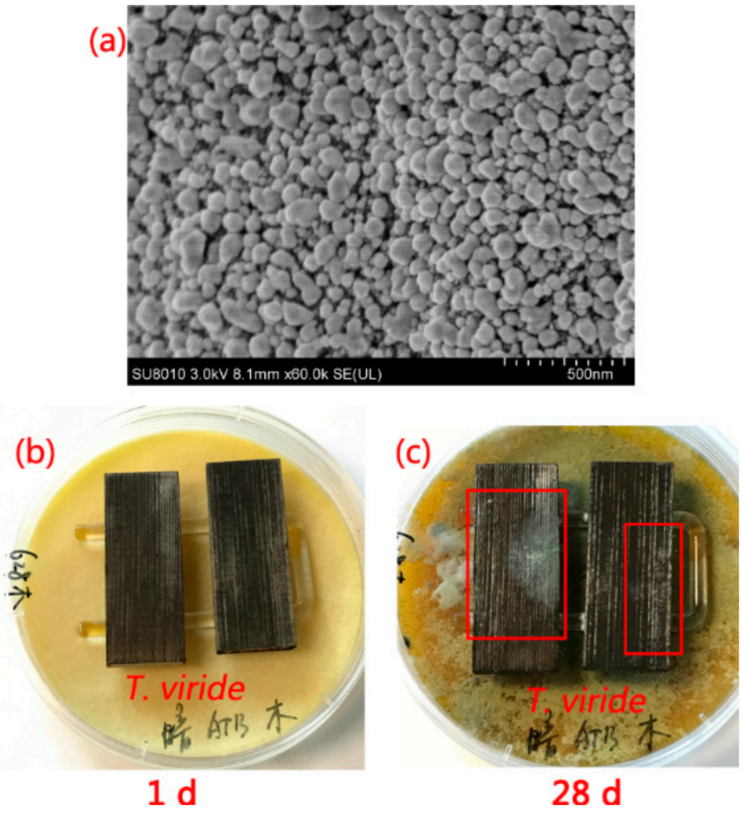

Figure 4. (**a**) SEM image of Ag/TiO_2/bamboo prepared with a silver mirror reaction. Antifungal property of Ag/TiO_2/bamboo to inhibit T. viride spores. (**b**) 1 day, (**c**) 28 days.

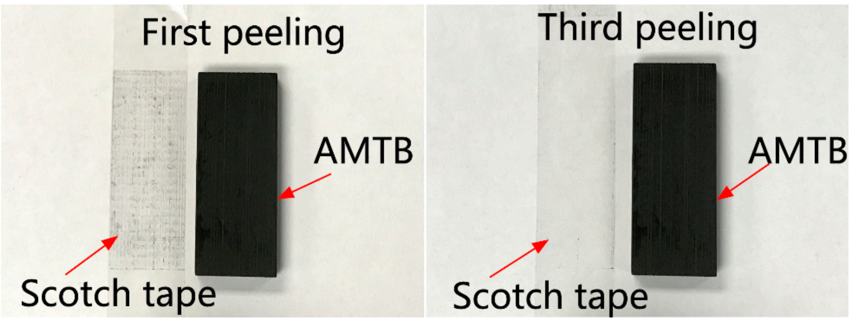

Figure S5. Optical images of AMTB sample after the first and third peeling tests.

Table S1. Summary of surface area and pore properties of AMTB sample.

Sample	S_{BET}/ m^2 g^{-1}	d_P/nm	V_P/cm^3 g^{-1}
MTB-4	65.4	2.5	0.04
AMTB	14.8	2.2	0.008

© 2019 by the authors. Licensee MDPI, Basel, Switzerland. This article is an open access article distributed under the terms and conditions of the Creative Commons Attribution (CC BY) license (http://creativecommons.org/licenses/by/4.0/).
